# Cross-reactive dengue human monoclonal antibody prevents severe pathologies and death from Zika virus infections

**DOI:** 10.1172/jci.insight.92428

**Published:** 2017-04-20

**Authors:** Yiu-Wing Kam, Cheryl Yi-Pin Lee, Teck-Hui Teo, Shanshan W. Howland, Siti Naqiah Amrun, Fok-Moon Lum, Peter See, Nicholas Qing-Rong Kng, Roland G. Huber, Mei-Hui Xu, Heng-Liang Tan, Andre Choo, Sebastian Maurer-Stroh, Florent Ginhoux, Katja Fink, Cheng-I Wang, Lisa F.P. Ng, Laurent Rénia

**Affiliations:** 1 Singapore Immunology Network, Agency for Technology and Research (A*STAR), Biopolis, Singapore.; 2 NUS Graduate School for Integrative Sciences and Engineering, National University of Singapore, Singapore.; 3 Bioinformatics Institute,; 4 Bioprocessing Technology Institute, A*STAR, Biopolis, Singapore.; 5 Department of Biomedical Engineering, Faculty of Engineering, National University of Singapore, Singapore.; 6 School of Biological Sciences, Nanyang Technological University, Singapore.; 7 Department of Biological Sciences, National University of Singapore, Singapore.; 8 Department of Biochemistry, Yong Loo Lin School of Medicine, National University of Singapore, Singapore.; 9 Institute of Infection and Global Health, University of Liverpool, United Kingdom.

## Abstract

Zika virus (ZIKV) infections have been linked with neurological complications and congenital Zika syndrome. Given the high level of homology between ZIKV and the related flavivirus dengue virus (DENV), we investigated the level of cross-reactivity with ZIKV using a panel of DENV human mAbs. A majority of the mAbs showed binding to ZIKV virions, with several exhibiting neutralizing capacities against ZIKV in vitro. Three of the best ZIKV-neutralizing mAbs were found to recognize diverse epitopes on the envelope (E) glycoprotein: the highly conserved fusion-loop peptide, a conformation-specific epitope on the E monomer, and a quaternary epitope on the virion surface. The most potent ZIKV-neutralizing mAb (SIgN-3C) was assessed in 2 type I interferon receptor–deficient (*IFNAR^–/–^*) mouse models of ZIKV infection. Treatment of adult nonpregnant mice with SIgN-3C rescued mice from virus-induced weight loss and mortality. The SIgN-3C variant with Leu-to-Ala mutations in the Fc region (SIgN-3C-LALA) did not induce antibody-dependent enhancement (ADE) in vitro but provided similar levels of protection in vivo. In pregnant ZIKV-infected *IFNAR^–/–^* mice, treatment with SIgN-3C or SIgN-3C-LALA significantly reduced viral load in the fetal organs and placenta and abrogated virus-induced fetal growth retardation. Therefore, SIgN-3C-LALA holds promise as a ZIKV prophylactic and therapeutic agent.

## Introduction

Zika virus (ZIKV) was first isolated in 1947 (1) and remained relatively neglected by the public health system for more than 50 years until its sudden reemergence in the Micronesian island of Yap in 2007 (2, 3). From there, the ZIKV epidemic continued to spread in several Pacific islands, finally reaching the Americas in 2015 (4–8). As of December 2016, 75 countries are experiencing ongoing transmission of ZIKV (9). While the symptoms of ZIKV infection are typically mild, there is now strong evidence linking ZIKV with Guillain-Barré syndrome (GBS) and congenital Zika syndrome (10–16). Thus, it continues to be a public health concern with severe social and economic impact. With no specific and licensed treatment available for ZIKV, there remains an urgent need to develop ZIKV prophylactic and therapeutic agents.

ZIKV is a flavivirus that is transmitted by *Aedes* mosquitoes. Typical symptoms include fever, arthritis/arthralgia, skin rash, conjunctivitis, joint pain, and headache (3, 17). Since the mode of transmission of ZIKV and the clinical symptoms caused by ZIKV infection are highly similar to that of dengue virus (DENV), distinguishing the 2 related flaviviruses in a clinical setting where both viruses are cocirculating can be challenging (18, 19). Conversely, the close antigenic relationship between ZIKV and DENV imply the existence of cross-reactive antibodies that are able to confer cross-protection against both viruses (20–24).

In order to characterize the cross-reactivity of human DENV mAbs against ZIKV, a validated panel of DENV-specific mAbs cloned from dengue patient plasmablasts was investigated (25, 26). We found 3 cross-reactive mAbs with the capacity to inhibit ZIKV infection in vitro that recognize different epitopes within the various ZIKV antigens. SIgN-3C, the mAb with the highest neutralizing capacity, was then assessed in both nonpregnant and pregnant murine models of ZIKV infection. Our results further strengthen the importance of antibody-mediated therapy in combating ZIKV.

## Results

### Broad ZIKV cross-reactivity by human DENV mAbs.

**Figure 1:**
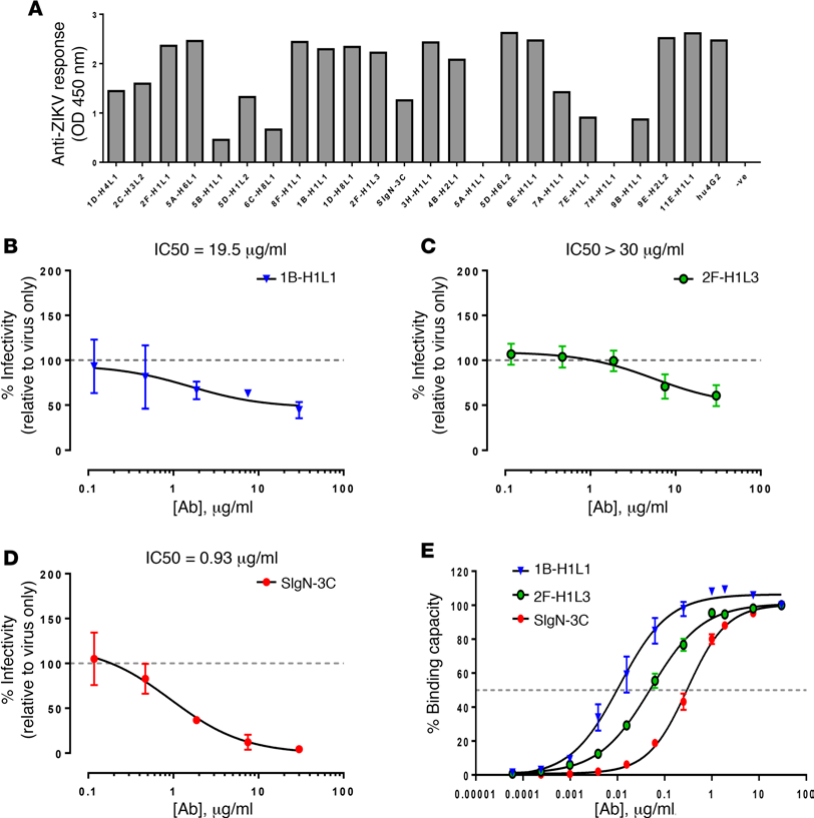
Binding and neutralizing activity of human DENV mAbs against Zika virus. (**A**) Level of recognition of Zika virus (ZIKV) whole virions by human dengue virus (DENV) mAbs was tested at 1 μg/ml (*n* = 3) and determined by ELISA using purified ZIKV virions. Data are presented as mean ± SD. (**B**–**D**) Neutralizing capacities of selected human DENV mAbs against ZIKV in vitro. ZIKV was preincubated with serial dilutions of human DENV mAbs 1B-H1L1 (**B**), 2F-H1L3 (**C**), or SIgN-3C (**D**) prior to infecting Vero-E6 cells at MOI of 10. Mock-infected and virus-only conditions were used as controls. Infectivity was quantified 48 hours after infection by immunofluorescence. Data are presented as mean ± SEM of 3 to 4 independent experiments, normalized to virus-only control. Nonlinear regression fitting was used to determine the IC50 values. (**E**) Binding curves of selected mAbs by ZIKV virion ELISA. OD values were normalized to the result at 30 μg/ml mAb.

Twenty-three human DENV mAbs that were cloned from the plasmablasts of 2 DENV-infected patients (25, 26) were assessed for cross-reactivity against ZIKV using virion-based ELISA assays. All but 2 of these human mAbs could recognize ZIKV whole virions at the concentration of 1 μg/ml (Figure 1A). This indicates a high level of cross-reactivity to ZIKV, consistent with the presence of structural similarities in the immunodominant regions between ZIKV and DENV (20, 21, 23).

### Some human DENV mAbs neutralize ZIKV.

The 23 human DENV mAbs were assessed for their ability to inhibit ZIKV infection of Vero-E6 cells (Supplemental Table 1; supplemental material available online with this article; https://doi.org/10.1172/jci.insight.92428DS1). While most of the antibodies were non-neutralizing at the concentrations tested, human mAbs 1B-H1L1, 2F-H1L3, and SIgN-3C showed considerable capacity to neutralize ZIKV in vitro (Figure 1, B–D). Based on the estimated antibody dose required for 50% neutralization (IC50), SIgN-3C demonstrated higher neutralizing activity (IC50 = 0.93 μg/ml) against ZIKV than 1B-H1L1 (IC50 = 19.25 μg/ml) and 2F-H1L3 (IC50 > 30 μg/ml). Furthermore, only SIgN-3C was able to approach complete neutralization at saturating concentrations, whereas the inhibition by 1B-H1L1 and 2F-H1L3 was incomplete (Figure 1, B–D), even though SIgN-3C demonstrated lower binding affinity for immobilized ZIKV virions than 1B-H1L1 and 2F-H1L3 (Figure 1E). The concentrations required for half-maximal binding to ZIKV virions were 0.011 μg/ml, 0.051 μg/ml, and 0.30 μg/ml for 1B-H1L1, 2F-H1L3, and SIgN-3C, respectively. Importantly, these 3 selected mAbs also showed binding and neutralizing activity against the Brazilian ZIKV PE243 strain (Supplemental Figure 1). Therefore, these human mAbs may be binding to different ZIKV epitopes that differ in accessibility or in their roles during infection.

### ZIKV antigens recognized by mAbs.

DENV envelope (E) glycoprotein, pre-membrane (prM) protein, and non-structural protein 1 (NS1) are the major antigenic targets in DENV infection (27, 28). To identify the ZIKV antigens recognized by the 3 human DENV mAbs with significant ZIKV neutralizing capacity, we created a panel of K562 cell lines transduced to express the ectodomain of ZIKV E, full-length ZIKV prM, and full-length ZIKV NS1 on the cell surface, fused to the transmembrane anchor of platelet-derived growth factor receptor. Cell surface display of the respective ZIKV antigens was confirmed by antibody labeling of epitope tags (Supplemental Figure 2). Each transduced cell line and control untransduced K562 cells were incubated with 1 μg/ml 1B-H1L1, 2F-H1L3, or SIgN-3C prior to detection with a fluorescent secondary antibody. K562 cells surface expressing ZIKV E were clearly bound by 1B-H1L1 and 2F-H1L3 (Supplemental Figure 3A), proving that these 2 antibodies recognize folded E glycoprotein. As expected, 1B-H1L1 and 2F-H1L3 also did not bind to cells surface-displaying ZIKV prM or NS1 (Supplemental Figure 3B). SIgN-3C at 1 μg/ml did not recognize any of the transduced cell lines, while at 10 μg/ml, there was a very low level of binding to cells surface-displaying ZIKV E (Supplemental Figure 3). Given that SIgN-3C bound well to whole ZIKV virions at 1 μg/ml (Figure 1E), we propose that this mAb recognizes a quaternary epitope on the virion surface, such as the interface of the E dimer, which has been described to be the epitope of a class of neutralizing human DENV mAbs (20).

### Epitope mapping and localization.

To identify any specific peptide regions responsible for antibody recognition, 2 overlapping peptide libraries of DENV and ZIKV E glycoproteins were synthesized. The 18-mer overlapping peptides (with an offset of 8 amino acids) were designed using the DENV3 consensus sequence and ZIKV Polynesian isolate sequence (Supplemental Figure 4). Peptide-based ELISA was first performed using pools of up to 5 consecutive peptides from each library (Supplemental Figure 5). 1B-H1L1 bound to several peptide pools from both DENV and ZIKV, whereas 2F-H1L3 gave low signals for all pools. Since 2F-H1L3 bound strongly to cells surface-displaying ZIKV E ectodomain (Supplemental Figure 3), the results suggest that this mAb recognizes a nonlinear conformational epitope on ZIKV E. Corroborating this hypothesis, an immunoblotting experiment on the lysate of cells expressing ZIKV E showed that 2F-H1L3 could not recognize the denatured E glycoprotein. SIgN-3C did not bind to any DENV or ZIKV linear peptides, which coincides with the hypothesis that this mAb is targeting a quaternary epitope on the DENV/ZIKV virion surface (20).

A second ELISA experiment was performed to test binding of 1B-H1L1 to the individual peptides from the positive pools (Supplemental Figure 6). 1B-H1L1 strongly recognized peptides from the fusion-loop regions of both DENV (Supplemental Figure 6A) and ZIKV (Supplemental Figure 6B), which are highly conserved (Supplemental Figure 6C). Identified epitope sequences were mapped onto available 3D crystal structures of the DENV E (Protein Data Bank [PDB] identifier 3J6U) and ZIKV E glycoprotein (PDB identifier 5IZ7) (29, 30) (Supplemental Figure 7). Spatial positioning on the dimeric E glycoprotein shows that the identified epitopes are located on exposed surface regions on the distal face of the E protein, accessible to antibody binding in the fully assembled viral particle.

### SIgN-3C mediates antibody-dependent infection enhancement.

**Figure 2:**
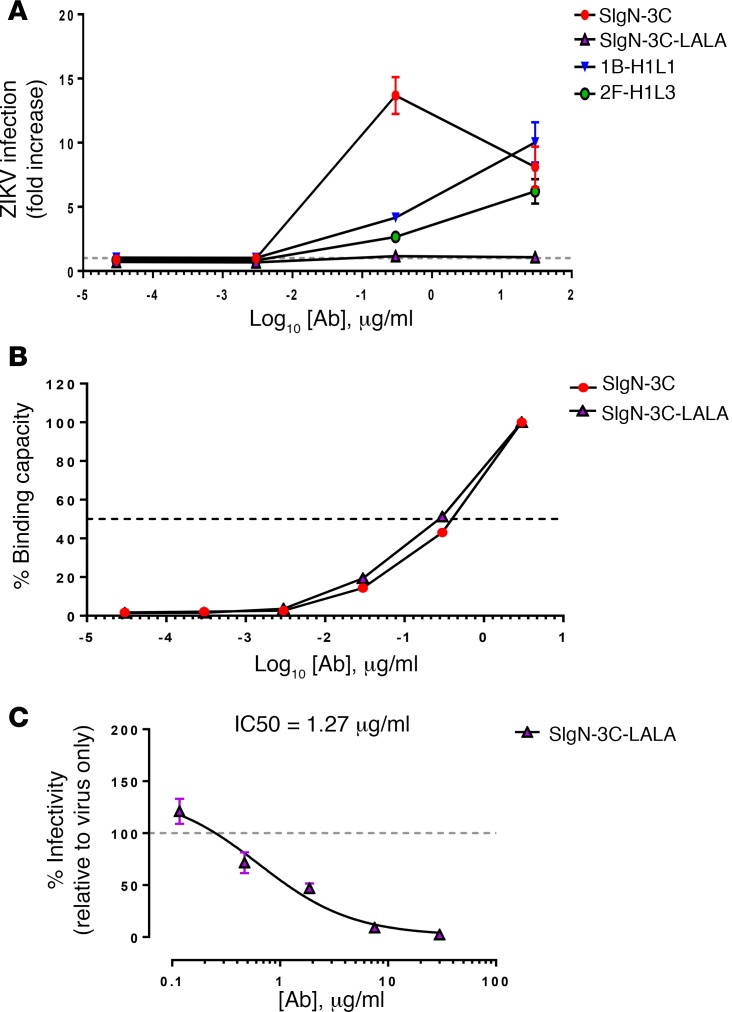
Modified SIgN-3C antibody (SIgN-3C-LALA) abrogated ADE in in vitro ZIKV infection. (**A**) Antibody-dependent enhancement (ADE) of Zika virus (ZIKV) infection. ZIKV was preincubated with serial dilutions of 1B-H1L1, 2F-H1L3, SIgN-3C, and SIgN-3C-LALA (0.03 ng/ml to 30 μg/ml) before infecting K562 cells at MOI of 10. Noninfected cells and virus infection in the absence of mAb (control infection, gray dotted line) were used as controls. Results are presented as mean ± SEM of virus titer fold increase with the presence of different concentrations of mAbs, relative to control infection. Results are presented as average of 2 independent experiments. (**B**) Binding curves of SIgN-3C and SIgN03C-LALA mAbs by ZIKV virion ELISA. OD values were normalized to the result at 3 μg/ml. (**C**) SIgN-3C-LALA antibody preserves good neutralizing activity against ZIKV. Data are presented as mean ± SEM of 4 independent experiments, normalized to virus-only control. Nonlinear regression fitting was used to determine the IC50 values.

Antibody-dependent infection enhancement (ADE) is a phenomenon that has been extensively studied in DENV infection in vitro and in vivo (31–35). To assess if ADE can occur during ZIKV infection, low to very low concentrations (30 μg/ml to 0.03 ng/ml) of 1B-H1L1, 2F-H1L3, and SIgN-3C mAb were tested using the ZIKV-ADE assay. A clear ADE phenomenon was observed, with a maximum fold increase in ZIKV viral load observed in the presence of 0.3 μg/ml of SIgN-3C (Figure 2A). The effect of ZIKV-ADE infection declined when lower SIgN-3C concentrations were used in the preincubation steps. To resolve the safety concern of using SIgN-3C as therapeutic antibody against ZIKV, the Fc mutant version of the 3C antibody (SIgN-3C-LALA) was generated by introducing Leu-to-Ala mutations in the Fc region that disrupt binding to Fc receptors (26, 36). SIgN-3C-LALA demonstrated similar ZIKV binding (Figure 2B) and neutralizing activity (Figure 2C), without enhancing ZIKV infection in vitro (Figure 2A).

### Therapeutic potential of SIgN-3C and SIgN-3C-LALA mAbs against ZIKV-induced mortality in nonpregnant IFNAR^–/–^ mice.

**Figure 3:**
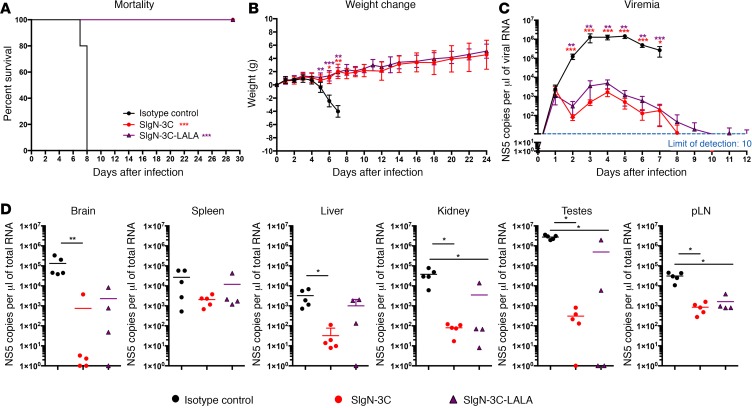
Treatment with SIgN-3C and its LALA variant prevent ZIKV-induced mortality in *IFNAR^–/–^* mice. (**A**) Mortality, (**B**) weight change, and (**C**) viremia of Zika virus–infected (ZIKV-infected) type I interferon receptor–deficient (*IFNAR^–/–^*) mice receiving isotype (*n* = 10), SIgN-3C (*n* = 5), and SIgN-3C-LALA (*n* = 10) antibodies. Mice were inoculated with 10^4^ PFU ZIKV s.c. at the ventral side of the footpad and treatments were given on days 1, 4, and 8 after infection. Mice were treated with 1 mg per dose of SIgN-3C and 0.5 mg per dose of SIgN-3C-LALA. Data shown are representative of 2 independent experiments. (**D**) Viral load in brain, spleen, liver, kidney, testes, and popliteal lymph node (pLN) of ZIKV-infected *IFNAR^–/–^* mice receiving isotype (*n* = 5), SIgN-3C (*n* = 5), and SIgN-3C-LALA (*n* = 4) antibodies at 6 days after infection. The mortality curve was analyzed using a log-rank (Mantel-Cox) test, while weight change, viremia, and viral load were analyzed using the Kruskal-Wallis test with Dunn’s multiple comparison. *P < 0.05; **P < 0.01; ***P < 0.001.

To assess the therapeutic potential of SIgN-3C and SIgN-3C-LALA, we employed an established mouse model using mice deficient for the type I interferon receptor (*IFNAR^–/–^*) (37). *IFNAR^–/–^* mice were infected subcutaneously (s.c.) with ZIKV, and SIgN-3C, SIgN-3C-LALA, or isotype control were administered intraperitoneally (i.p.) at 1, 4, and 8 days postinfection (dpi). Virus-infected mice injected with the isotype control showed symptoms of weakness with rapid weight loss from 4 dpi onwards and all died on 7 to 8 dpi (Figure 3, A and B). In contrast, virus-infected mice treated with either SIgN-3C or the LALA variant did not display any signs of illness and continued to gain weight until they were euthanized at 4 weeks after treatment (Figure 3, A and B). Survival of these animals was due to the control of virus replication in both the circulatory system and the major organs (Figure 3, C and D). Both treatments suppressed peak viremia from 3 to 7 dpi by 3 to 4 orders of magnitude, and cleared viremia by 12 dpi (Figure 3C). Importantly, at 6 dpi when control mice displayed severe ZIKV-induced symptoms prior to mortality at 7 dpi, viral loads in the brain, liver, kidney, testes, and popliteal lymph nodes (pLNs) were significantly reduced in mAb-treated mice (Figure 3D). These data suggest that treatment with SIgN-3C or its LALA variant prevented ZIKV-induced mortality in *IFNAR^–/–^* animals with a comparable degree of protective efficacy.

### SIgN-3C and SIgN-3C-LALA protect fetuses from ZIKV-induced growth retardation in pregnant IFNAR^–/–^ mice.

**Figure 4:**
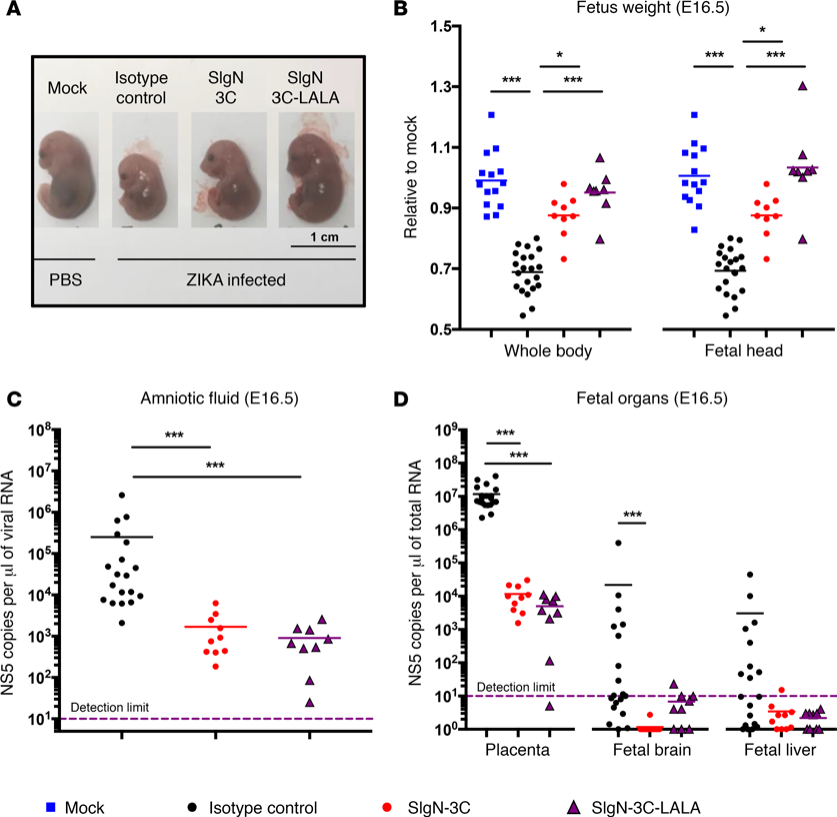
SIgN-3C and SIgN-3C-LALA treatments prevent ZIKV-induced congenital developmental deficiency in *IFNAR^–/–^* mice. (**A**) Representative images and (**B**) weight of fetuses isolated from mock-infected type I interferon receptor–deficient (*IFNAR^–/–^*) mice, Zika virus–infected (ZIKV-infected) isotype control, and ZIKV-infected mice with SIgN-3C or SIgN-3C-LALA treatments (all groups *n* ≥ 7). Each data point in dot plots was obtained from 1 fetus. Weights of fetuses are expressed relative to the mean of fetal weights from mock-infected *IFNAR^–/–^* mice. Viral load in the (**C**) amniotic fluid and (**D**) organs of fetuses from ZIKV-infected *IFNAR^–/–^* pregnant mice receiving isotype, SIgN-3C, and SIgN-3C-LALA antibodies (all groups *n* ≥ 7). Each data point in dot plots was obtained from 1 fetus. Mice were inoculated with 10^7^ PFU ZIKV i.v. on E10.5 and treatments were given on days 0, 1, and 3 after infection. Mice were given 0.5 mg of isotype or treatment antibody per dose. All animals were harvested at E16.5. All data were analyzed using the Kruskal-Wallis test with Dunn’s multiple comparison. *P < 0.05, ***P < 0.001.

ZIKV infection of pregnant mice deficient in type I interferon signaling has recently been reported to result in fetal growth restriction (38, 39). We next explored the potential of SIgN-3C and SIgN-3C-LALA to protect *IFNAR^–/–^* fetuses from ZIKV-induced congenital pathology. Pregnant *IFNAR^–/–^* mice infected with a high virus titer (10^7^ PFU) at E10.5 resulted in growth retardation in the fetuses with high levels of viral RNA (vRNA) detected in the amniotic fluid, placenta, and fetal brain at E16.5 (Figure 4). vRNA was detectable in the fetal liver at E16.5 at a much lower titer (Figure 4D). Notably, fetuses from virus-infected and isotype-treated mice were smaller in size and had lower whole-body and head mass compared with fetuses from mock-infected mice at E16.5 (Figure 4, A and B). However, administration of SIgN-3C or SIgN-3C-LALA at 0, 1, and 3 dpi restored head and whole-body mass of fetuses from virus-infected animals to values similar to those from noninfected mice (Figure 4, A and B). Protection from ZIKV-induced congenital developmental deficiency was also associated with lower viral loads in the amniotic fluid (Figure 4C), placenta, fetal brain, and fetal liver in the treated mice (Figure 4D). Both the fetal weight and viral load in the various organs tested were similar between SIgN-3C–treated and SIgN-3C-LALA–treated mice (Figure 4).

## Discussion

Given the greater than 50% protein sequence identity between DENV and ZIKV, it is not surprising that almost all of the human DENV mAbs studied had some level of cross-reactivity with ZIKV. While we were concluding this work, other groups also reported a high level of ZIKV cross-reactivity by DENV antibodies or sera from DENV patients (20–23). Here, we identified and characterized 3 DENV mAbs with neutralizing activity against ZIKV, with 3 different modes of binding to ZIKV E glycoprotein. 1B-H1L1 recognizes a linear epitope, the conserved fusion-loop region of DENV and ZIKV. 2F-H1L3 recognizes a nonlinear conformational epitope present on the folded E glycoprotein monomer, which we expressed on the K562 cell surface. Despite binding to whole ZIKV virions, SIgN-3C had very weak binding to E glycoprotein monomer. Barba-Spaeth et al. categorized their DENV/ZIKV cross-reactive antibodies into those targeting the fusion-loop epitope and those targeting the E-dimer epitope (21). 3C-H5L1 may belong to the latter category.

While Barba-Spaeth et al. found that their cross-reactive fusion loop–binding mAbs had poor ZIKV neutralizing capacity even at 1 μM (~150 μg/ml) (21), our fusion-loop binder 1B-H1L1 clearly decreased ZIKV infectivity at 2 μg/ml onwards. With an IC50 of 19.25 μg/ml, SIgN-3C is also considerably more potent at neutralizing ZIKV in vitro than the murine ZIKV/DENV fusion loop–targeting antibody 2A10G6, which had a 50% plaque reduction titer of 249 μg/ml (24). On the other hand, SIgN-3C is not as potent as antibodies that were isolated from ZIKV-immunized mice (40) or ZIKV-infected patients (41–43), which had reported IC50 values as low as 0.1 and 0.01 μg/ml, respectively. However, it is difficult to compare IC50 values obtained using different methodologies and virus strains, and ultimately, in vivo efficacy is more important. As demonstrated in our current animal study, SIgN-3C was effective in suppressing ZIKV viremia, thereby protecting mice from rapid weight loss and ZIKV-induced lethality seen in the untreated group at 7 to 8 dpi.

Arguably, the largest health threat posed by the ZIKV epidemic is the risk of microcephaly and other fetal abnormalities in virus-infected pregnant women. Recent studies have shown that in mice lacking type I interferon signaling, ZIKV infection of pregnant mice resulted in virus transmission across the placenta to the fetus, causing fetal growth restriction and brain damage (38, 39). Here, we provide a proof of principle in a similar pregnant mouse model that maternal administration of SIgN-3C or SIgN-3C-LALA can result in drastic reduction of viral load in the placenta and developing fetus and thus improve fetal development. Taken together with the work of Sapparapu et al. using a mAb isolated from a ZIKV patient (42), our work supports the possibility of using neutralizing antibodies as prophylactic or therapeutic interventions for pregnant women in ZIKV-endemic areas.

Repurposing of currently available mAbs against different pathogens can not only shorten the development time of new therapeutics but also broaden the therapeutic spectrum of the same mAb. Similar strategies have been proposed from various studies (24, 44). However, further antibody engineering of SIgN-3C may be vital for improving the safety and efficacy of this potential ZIKV therapeutic. As this mAb was induced against DENV and not ZIKV, it is likely that its potency can be improved by antibody affinity maturation using a directed evolution approach towards better ZIKV virion recognition. ADE of ZIKV infection by DENV antibodies remains a significant concern (20, 22). In flaviviruses, ADE-mediated DENV infection has been suggested to cause severe clinical outcomes following secondary DENV infections (45, 46) due to the cross-reactivity between DENV-specific antibodies and different serotypes of DENV (33). Hypotheses have suggested that the occurrence of extrinsic and intrinsic ADE mechanisms (33) cause high circulating virus titers in patients that lead to severe clinical outcomes . For a therapeutic mAb, there is a risk of ADE when its serum concentration drops below its neutralizing threshold. Improving the human mAb affinity is likely to lower its neutralization threshold and thus reduce the risk of ADE. Engineering the Fc region of the human mAb to reduce Fc receptor binding has been shown to abrogate ADE by DENV antibodies (41, 47, 48). We show in this study that introducing the LALA mutation in SIgN-3C eliminated ZIKV ADE in vitro without compromising the neutralization capacity in vivo, presenting SIgN-3C-LALA as a viable alternative that sufficiently protects against ZIKV infection in vivo. On the other hand, a possible link between the Sanofi *Dengvaxia* vaccine and patients who displayed severe clinical outcomes could be coupled with the presence of cross-reactive antibodies that induce ADE, and also the lack of protective T cell immunity after vaccination (49). A recent study has indicated the importance of DENV NS1 protein–mediated T cell response in natural immunity or vaccination to confer protection against DENV infection (50). Therefore, we recommend that comprehensive protection from ZIKV infection might require the presence of antibodies (for example SIgN-3C-LALA) in the vaccine formulation and the eventual activation of T cell immunity through presentation of ZIKV NS1 protein.

## Methods

### Virus.

The ZIKV Polynesian isolate was obtained from the European Virus Archive (EVA) and the ZIKV Brazilian isolate was obtained from the Centre for Virus Research, University of Glasgow, Glasgow, UK. Viruses were propagated in Vero-E6 cells (ATCC, CRL-1587) or in C6/36 cells (ATCC, CRL-1660) and prepared for infection studies. Expanded virus stocks were purified via ultracentrifugation (51), and titered by standard plaque assay using Vero-E6 cells (52).

### Expression and purification of human DENV mAbs.

Single plasmablasts were isolated from 2 DENV-infected patients (1 DENV2- and 1 DENV3-infected patient) and human DENV mAbs were expressed and purified from HEK-293-6E cells as described previously (25, 26).

### Virus-neutralization assay.

Neutralizing activity of human DENV mAbs was tested in triplicate and analyzed by immunofluorescence-based cell infection assay in Vero-E6 cells. ZIKV were mixed at MOI 10 with diluted (0.029–30 μg/ml) human mAb and incubated for 2 hours at 37°C with gentle agitation (350 rpm). Virus-mAb mixtures were then added to Vero-E6 cells seeded in 96-well plates and incubated for 2 hours at 37°C. Medium was removed, and cells were replenished with DMEM (Hyclone) supplemented with 10% FBS (Hyclone) and incubated for 48 hours at 37°C before fixation with 4% paraformaldehyde (Electron Microscopy Sciences). Cells were permeabilized with PBS containing 0.2% Tween-20 and incubated for 10 minutes at room temperature. Cells were stained with mouse anti-flavivirus antibody (Merck Millipore, clone D1-4G2-4.15) diluted in PBS for 1 hour at 37°C. This was followed by incubation with goat anti-mouse secondary antibody conjugated to fluorescein isothiocyanate (FITC) (Life Technologies; catalog F-2761) for 1 hour at 37°C. Cell nuclei were labeled with DAPI. Images were acquired and analyzed quantitatively by the Cellomics ArrayScan VTI HCS Reader. IC50 was calculated with Prism software (GraphPad Software, Inc.), applying a 3-parameter nonlinear regression curve fit.

### Human mAb reactivity with ZIKV determinants.

First, a ZIKV virion-based ELISA assay was performed as described previously (51). Virion-reactive human mAbs were then tested for their capacity to react with ZIKV single antigens expressed on the surface of transfected K562 cells. Using a synthetic codon-optimized sequence based on the published Suriname isolate (KU312312, see ref. 7), the ectodomain of ZIKV E (with the transmembrane region deleted) was cloned into the pDisplay vector (Life Technologies), which fuses the protein to an N-terminal signal peptide and a C-terminal platelet-derived growth factor receptor transmembrane anchor. The complete open reading frame was then subcloned into the lentiviral transfer vector pWPXL (provided by Didier Trono, Ecole Polytechnique Fédérale de Lausanne, Lausanne, Switzerland). Lentiviral particles were generated as previously described (53) and used to transduce K562 cells, which were then sorted based on expression of the HA epitope tag. Similar K562 cell lines surface-displaying ZIKV prM and NS1 were generated using lentivector constructs bearing full-length synthetic genes. Surface expression of the ZIKV E ectodomain was greatly enhanced by incubating the cells at 32°C for 24 hours. Each cell line was then incubated with 1 or 10 μg/ml of human DENV mAb for 30 minutes at room temperature prior to labeling with an Alexa Fluor 647 goat anti-human secondary antibody (ThermoFisher Scientific; catalog A-21445) for analysis by flow cytometry.

### Peptide-based ELISA.

Epitope screening was performed on synthesized biotinylated peptides (Mimotopes) consisting of 18-mer overlapping peptides generated from ZIKV Polynesian isolate (KJ776791) and the consensus sequence of DENV3 strains (KR296743, KF973487, EU081181, KF041254, JF808120, JF808121, KJ189293, KC762692, KC425219, KJ830751, KF973479, and AY099336) as previously described (51).

### Protein structures.

Structure of the dengue serotype 3 and Zika E proteins were retrieved from the PDB (54). The dengue serotype 3 E structure was derived from the cryo-EM structure PDB 3J6U (29) and the full atomic representation was reconstructed using Yasara (55). The Zika E structure is derived from PDB 5IZ7 (30). All structures were visualized using PyMOL.

### ZIKV-ADE assay.

ZIKV (MOI 10) was first incubated with diluted human DENV mAbs in serum-free IMDM (Gibco) for 2 hours on a shaking heat block (37°C, 350 rpm) before being used to infect the K562 cells for 1.5 hours in a 37°C incubator, supplemented with 5% CO_2_. The virus overlay was removed and cells were washed once before they were resuspended in appropriate complete medium. Cells were further incubated at 37°C, supplemented with 5% CO_2_. Seventy-two hours after infection, 140 μl of infected cell suspension was aliquoted for vRNA extraction. Mock and nonenhanced control infections were performed by incubating cells with serum-free IMDM and viruses, respectively. Results are expressed as fold increase of ZIKV viral load relative to nonenhanced infections.

### Mouse protection experiments.

For the nonpregnant ZIKV mouse model, 4-week-old *IFNAR^–/–^* mice in the C57BL/6J background were used. Animals were inoculated s.c. in the ventral side of the right footpad with 10^4^ PFU ZIKV propagated in C6/36 cells (30). Mice were subsequently given 500 μg to 1 mg of human DENV mAb SIgN-3C or SIgN-3C-LALA via i.p. injection on 1, 4, and 8 dpi. A human chimeric IgG1 isotype control with an unrelated specificity was included as isotype control group. Mortality, weight loss, disease signs, viremia, and viral load in major organs were monitored.

For the pregnant ZIKV mouse model, 8- to 10-week-old female *IFNAR^–/–^* mice were used for time mating and infection. Particularly, mating pairs housed in the same cage were separated in the morning and embryonic development was estimated by considering the day of vaginal plug observation as 0.5 days after conception. Pregnant mice were inoculated intravenously (i.v.) with 10^7^ PFU ZIKV on day 10.5 after conception. Mice were subsequently given 500 μg of SIgN-3C-LALA or isotype control mAb via i.p. injection on 0, 1, and 3 dpi. On day 16.5 after conception, pregnant mice were sacrificed and fetuses were harvested. Fetus weights were taken and viral load in the amniotic fluid, placenta, fetal brain, and fetal liver were quantified.

### Viral load quantification.

For viremia quantification, 10 μl of blood collected from the tail vein was diluted in 120 μl PBS and 10 μl citrate-phosphate-dextrose solution (Sigma-Aldrich). vRNA was extracted with a QIAmp Viral RNA kit (QIAGEN) in accordance with the manufacturer’s protocol. For viral load quantification in amniotic fluid, total amniotic fluid was collected from each conceptus sac after disruption of the surrounding membrane. vRNA was extracted with a QIAmp Viral RNA kit. For all other solid tissues, samples were placed in 2-ml microtubes (Labcon) containing 500 μl to 1 ml of TRIzol (Invitrogen) and 2-mm disruption beads (Tomy, model ZB-20). Tissues were homogenized twice in a bead-based cell disrupter (Micro Smash MS-100) at 5,000 rpm for 45 seconds each. Total RNA was then extracted into the aqueous phase using a TRIzol-chloroform method as previously described (56), followed by purification using an RNeasy kit (QIAGEN) following the manufacturer’s protocol. NS5 RNA copies were quantified in 1 μl of total RNA or vRNA by quantitative reverse transcription PCR (qRT-PCR) using the QuantiTect Probe RT-PCR Kit (QIAGEN) adapted from a previously reported protocol (57).

### Statistics.

All data were analyzed using Prism software. For virus-neutralization assays, the data were curve-fit by 3-parameter nonlinear regression analysis, after which the mAb dose that resulted in 50% neutralization was calculated (IC50). For all data presented in the animal studies, Kruskal-Wallis with Dunn’s multiple comparisons were done for datasets with 3 groups and above, while Mann-Whitney 2-tailed analysis was done for datasets with 2 groups. All in vitro data are presented as mean ± standard deviation (SD) or standard error of the mean (SEM); in vivo data are presented as mean (1 dot per sample). *P* values less than 0.05 were considered statistically significant.

### Study approval.

All mouse studies were approved by the IACUC (approval 151038) of the Singapore A*STAR, performed according to the guidelines of the Agri-Food and Veterinary Authority and the National Advisory Committee for Laboratory Animal Research of Singapore. All animals used were bred under specific pathogen–free conditions in the Biological Resource Centre (A*STAR).

## Author contributions

YWK, CLYP, THT, SWH, SNA, FML, PS, and NQRK performed immunological, virological, biochemical cell-based assays, and animal studies. MHX, KF, and CIW provided the human DENV mAbs. RGH and SMS performed the structural localization maps. HLT and AC generated the chimeric human IgG1 isotype antibody. YWK, CLYP, THT, SWH, FG, LFPN, and LR conceptualized the study. YWK, CLYP, SWH, SNA, FML, THT, LR, and LFPN analyzed the data. YWK, SWH, CLYP, THT, LR, and LFPN wrote the manuscript. All authors read and approved of the manuscript.

## Supplementary Material

Supplemental data
