# Supplementary material for: Cross-reactive dengue human monoclonal antibody prevents severe pathologies and death from Zika virus infections
Source: JCI Insight. 2017 Apr 20;2(8):e92428. doi: 10.1172/jci.insight.92428 (PMC5396524; doi:10.1172/jci.insight.92428)
Supplement: Supplemental data [file jciinsight-2-92428-s001.pdf]

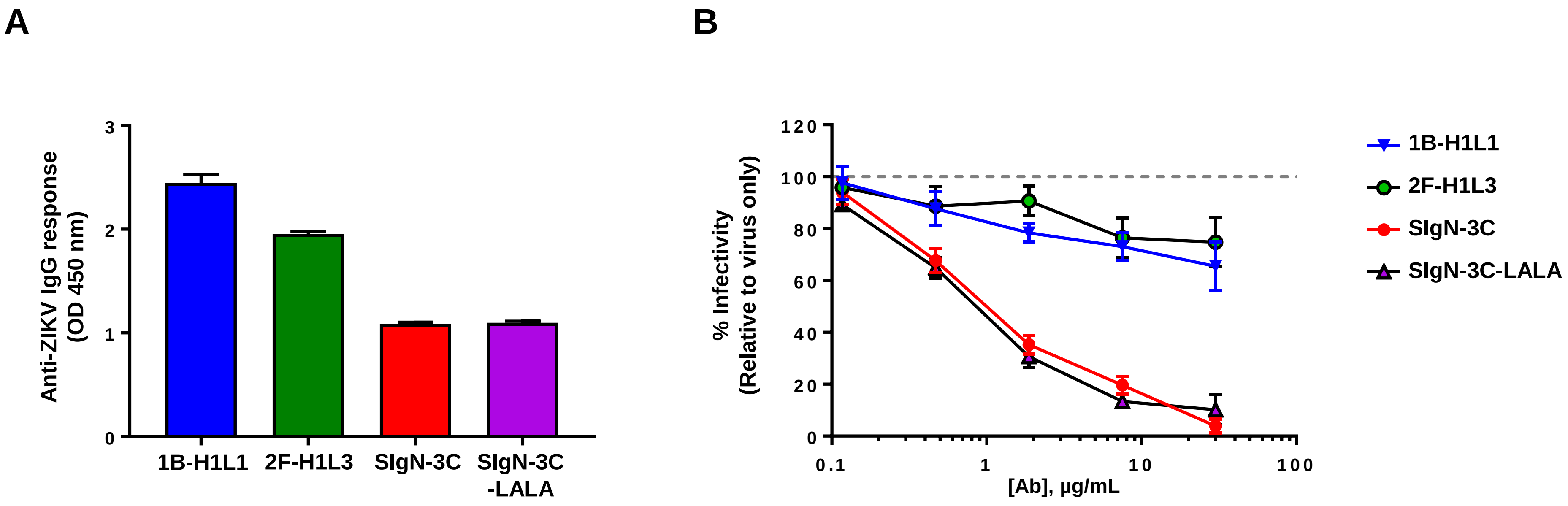

Supplemental Figure 1

Binding and neutralizing activity of human DENV monoclonal antibodies against the Brazilian ZIKV PE243 strain. (**A**) Level of recognition of ZIKV whole virions by human DENV mAbs was all tested at 3 µg/mL (n = 3) and determined by ELISA using purified ZIKV PE243 strain virions. Data are presented as mean ± SD. (**B**) Neutralizing capacities of selected human DENV mAbs against the Brazilian ZIKV PE243 strain in *vitro*. ZIKV PE243 strain was pre-incubated with serial dilutions of human DENV mAbs 1B-H1L1, 2F-H1L3, SIgN-3C, and SIgN-3C-LALA prior to infecting Vero-E6 cells at MOI of 10. Mock-infected and virus-only conditions were used as controls. Infectivity was quantified 48 h post-infection by immunofluorescence. Data are presented as mean ± SEM of 3 independent experiments, normalized to virus-only control.

Supplemental Figure 1 Kam *et al*., 2017

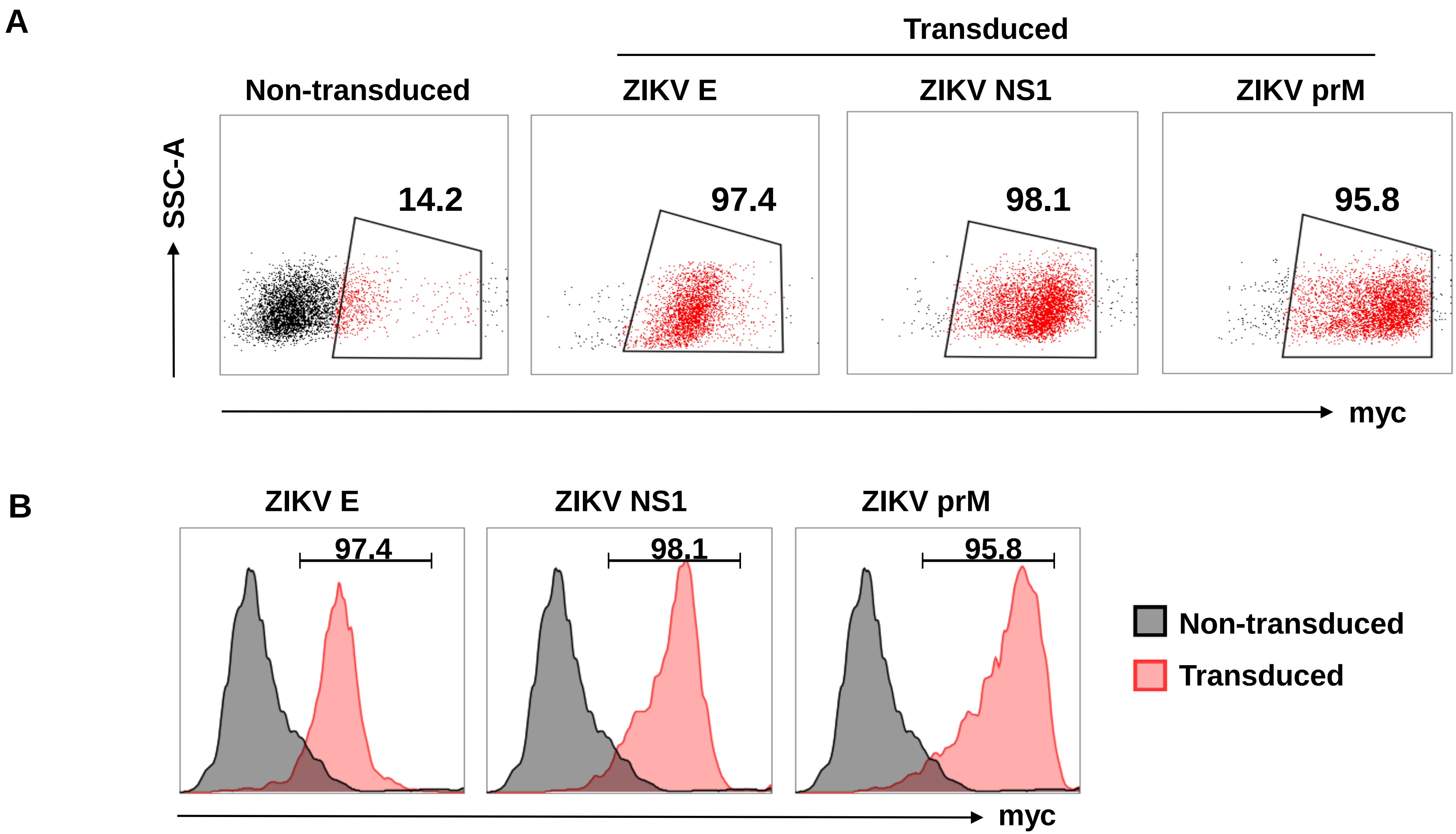

Supplemental Figure 2

Successful generation and expression of ZIKV antigens on K562 cell surface. Non-transduced K562 cells and K562 cells surface-displaying the ZIKV E ectodomain, NS1 or prM were labeled with anti-rabbit myc antibody. An Alexa Fluor 647-conjugated goat anti-rabbit secondary antibody was used to quantify binding by flow cytometry. (**A**) Dot plots showing the percentage expression of myc. Gates were drawn based on the secondary only control for each cell line. (**B**) Red histograms of transduced K562 cells are overlaid over grey histograms of control untransduced K562 cells. Results are representative of 3 independent experiments.

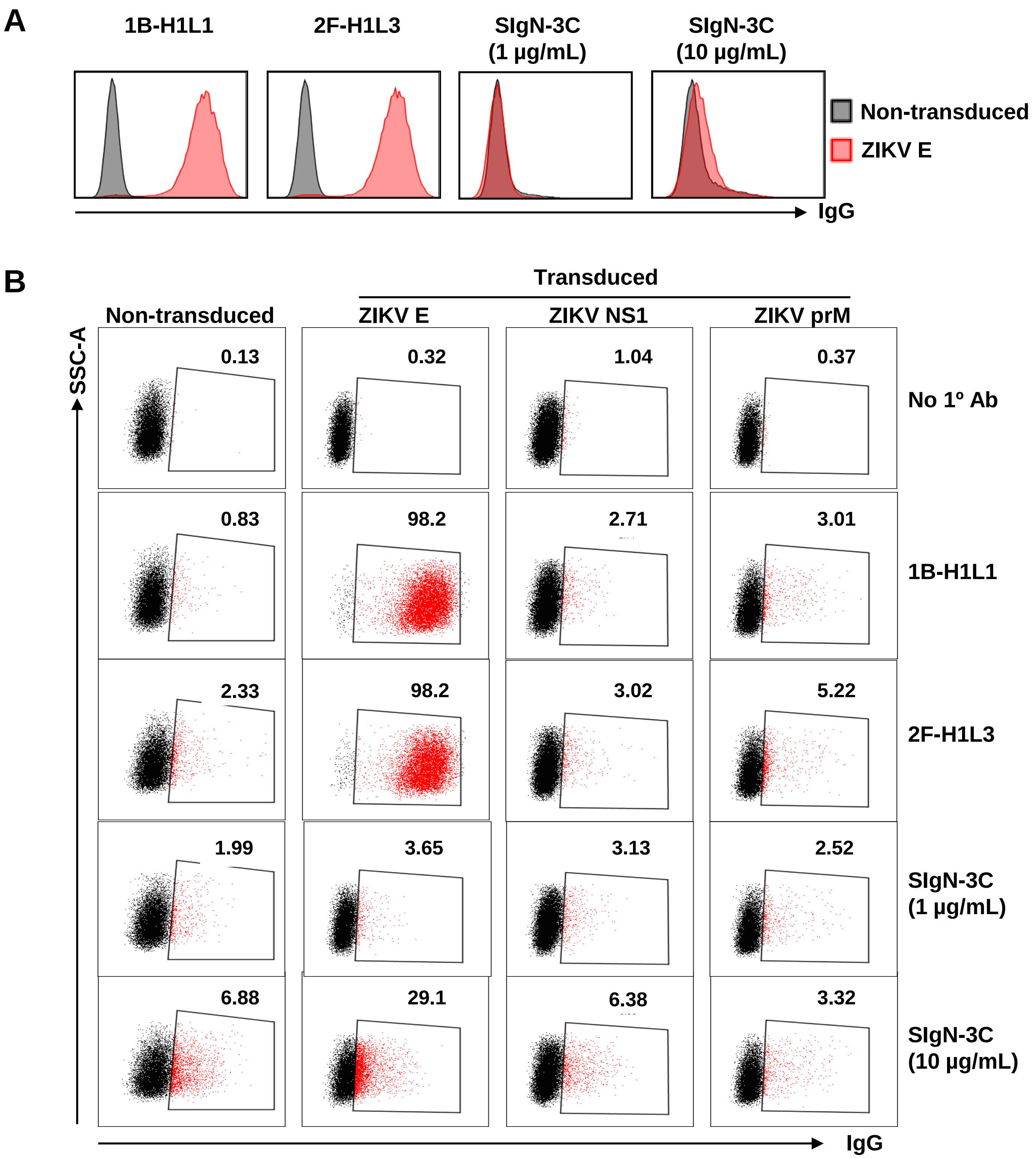

Supplemental Figure 3

Binding of mAbs to cell surface-expressed ZIKV antigens. Untransduced K562 cells and K562 cells surface-displaying the ZIKV E ectodomain, prM or NS1 were labeled with each mAb at 1 µg/mL. SIgN-3C was also tested at 10 µg/mL. An Alexa Fluor 647-conjugated secondary antibody was used to quantify binding by flow cytometry. (**A**) Red histograms of K562-ZIKV E cells are overlaid over grey histograms of control untransduced K562 cells. (**B**) Gates were drawn based on the secondary only control for each cell line. Results are representative of 3 independent experiments.

Supplemental Figure 3 Kam *et al*., 2017

E glycoprotein

```
ZIKV H/PF/2013 1    IRCIGVSNRDFVEGMSGGTWVDVVLEHGGCVTVMAQDKPTVDIELVTTTVSNMAEVRSYC
DENV3          1    MRCVGVGNRDFVEGLSGATWVDVVLEHGGCVTTMAKNKPTLDIELQKTEATQLATLRKLC

               61   YEASISDMASDSRCPTQGEAYLDKQSDTQYVCKRTLVDRGWGNGCGLFGKGSLVTCAKFA
               61   IEGKITNITTDSRCPTQGEAVLPEEQDQNYVCKHTYVDRGWGNGCGLFGKGSLVTCAKFQ

               121  CSKKMTGKSIQPENLEYRIMLSVHGSQHSGMIVNDTGHETDENRAKVEITPNSPRAEATL
               121  CLEPIEGKVVQYENLKYTVIITVHTGDQH-----QVGNETQG--VTAEITPQASTTEAIL

               181  GGFGSLGLDCEPRTGLDFSDLYYLTMNNKHWLVHKEWFHDIPLPWHAGADTGTPHWNNKE
               174  PEYGTLGLECSPRTGLDFNEMILLTMKNKAWMVHRQWFFDLPLPWTSGATTETPTWNRKE

               241  ALVEFKDAHAKRQTVVVLGSQEGAVHTALAGALEAEMDGAKGRLSSGHLKCRLKMDKLRL
               234  LLVTFKNAHAKKQEVVVLGSQEGAMHTALTGATEIQNSGGTS-IFAGHLKCRLKMDKLEL

               301  KGVSYSLCTAAFTFTKIPAETLHGTVTVEVQYAGTDGPCKVPAQMAVDMQTLTPVGRLIT
               293  KGMSYAMCTNTFVLKKEVSETQHGTILIKVEYKGEDAPCKIPFSTE-DGQGKAHNGRLIT

               361  ANPVITESTENSKMMLELDPPFGDSYIVIGVGEKKITHHWHRSGSTIGKAFEATVRGAKR
               352  ANPVVTKKEE--PVNIEAEPPFGESNIVIGIGDNALKINWYKKGSSIGKMFEATARGARR

               421  MAVLGDTAWDFGSVGGALNSLGKGIHQIFGAAFKSLFGGMSWFSQILIGTLLMWLGLNTK
               410  MAILGDTAWDFGSVGGVLNSLGKMVHQIFGSAYTALFSGVSWVMKIGIGVLLTWIGLNSK

               481  NGSISLMCLALGGVLIFLSTAVSA
               470  NTSMSFSCIAIGIITLYLGAVVQA
```

Supplemental Figure 4

Sequence alignment of the E glycoprotein from DENV and ZIKV. The consensus sequence of DENV3 and ZIKV Polynesia isolate (ZIKV H/PF/2013 – GenBank ID: KJ776791) used in the peptide library synthesis. Residues that are conserved between the DENV and ZIKV sequence are highlighted in red.

Supplemental Figure 4 Kam *et al*., 2017

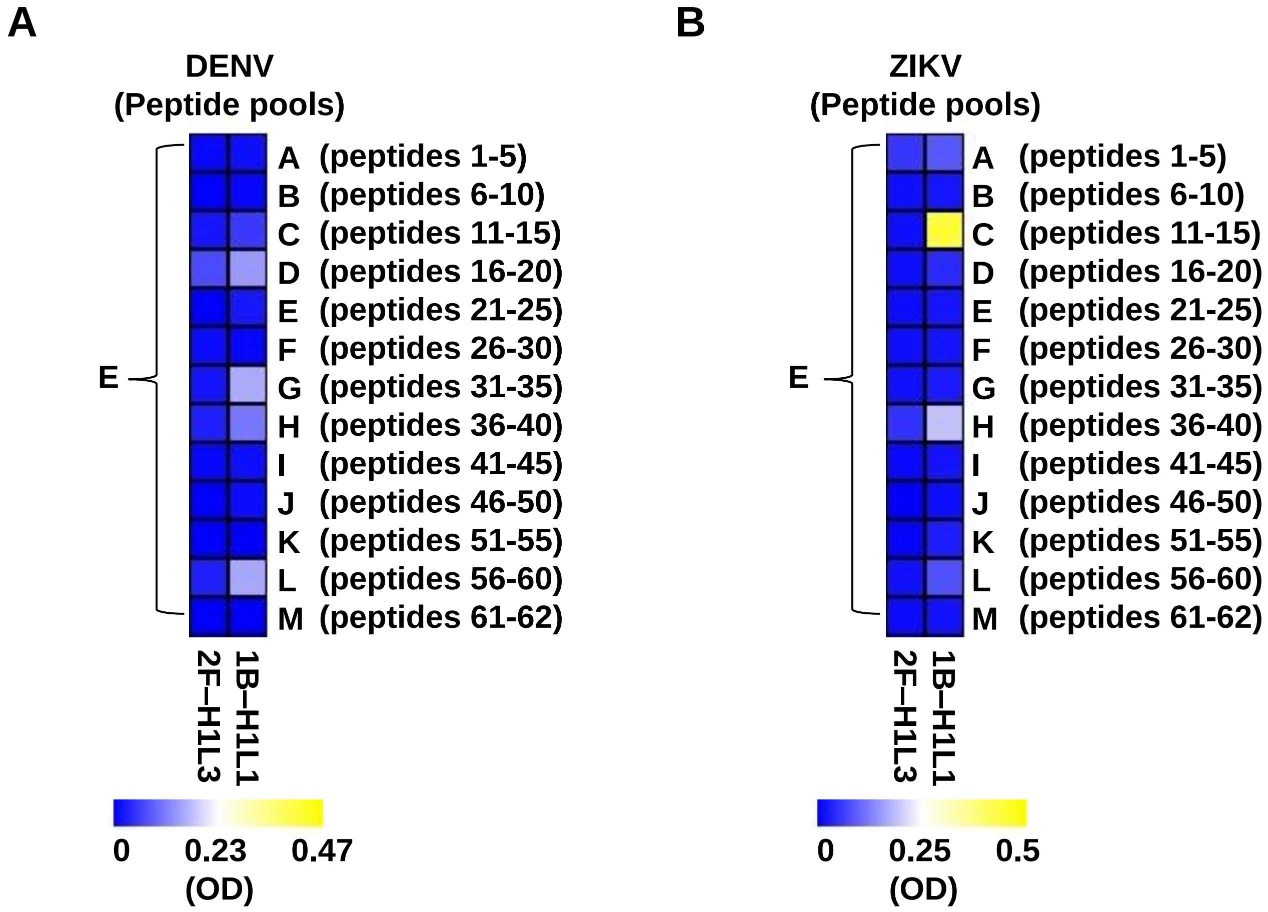

Supplemental Figure 5

ELISA using peptide pools from DENV E (**A**) and ZIKV E (**B**) overlapping peptide libraries. Peptides from each library were grouped into 13 pools (A-M) as indicated to perform ELISA assays. Each mAb was tested at 1 µg/ml. Selected human DENV monoclonal antibodies were subjected to (**A**) DENV (DENV peptide pools A – M) and (**B**) ZIKV (ZIKV peptide pools A – M) peptide-based ELISA assays corresponding to the DENV and ZIKV E glycoproteins. Data are presented in heat-map format with blue representing no binding and yellow color representing the highest OD. Results represent an average of two independent experiments.

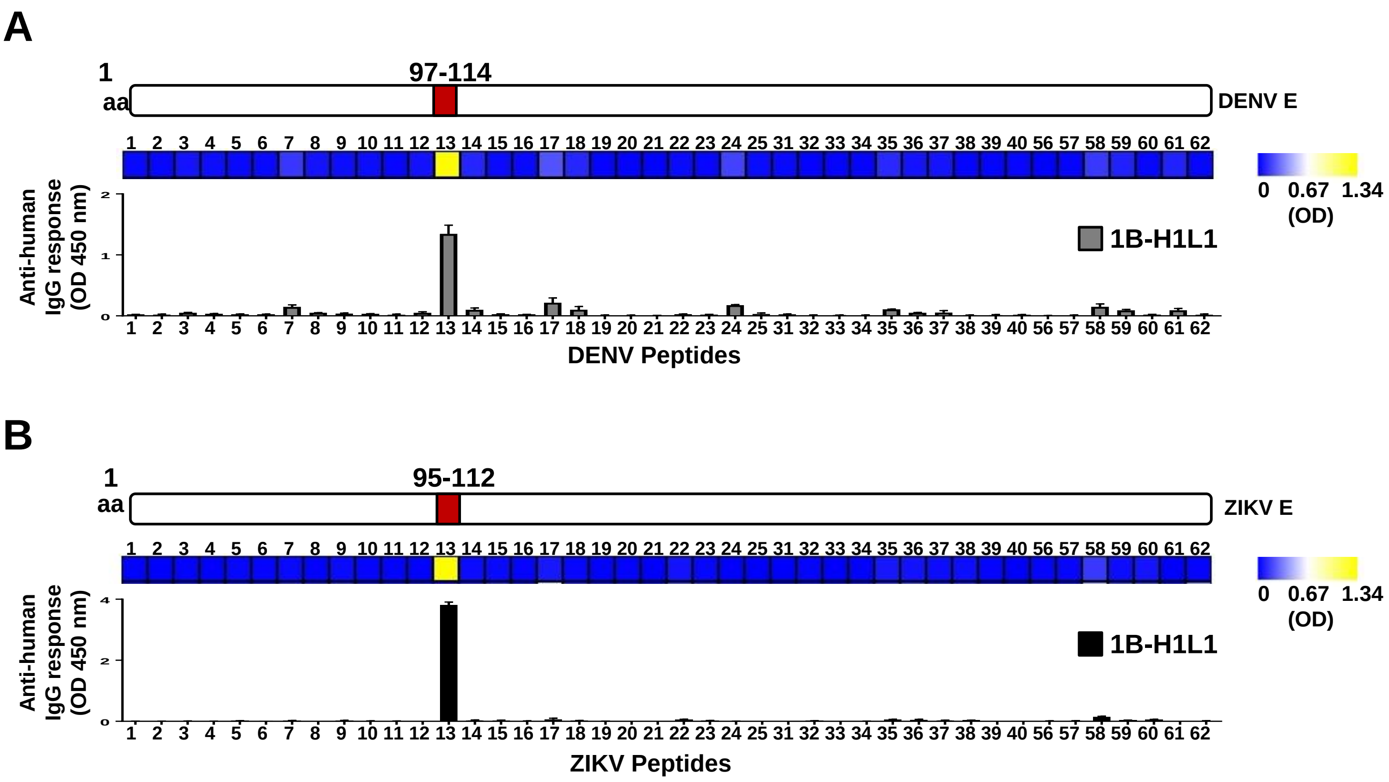

C

	DENV E		ZIKV E	
Antibody	Peptide sequence	Amino acid	Peptide sequence	Amino acid
1B-H1L1	VDRGWGNGCGLFGKGSLV	97-114	TLVDRGWGNGCGLFGKGS	95-112

Supplemental Figure 6

Linear epitope mapping on ZIKV E glycoprotein. (**A** and **B**) 1B-H1L1 was tested for binding to selected DENV E and ZIKV E peptides by ELISA at 1 ug/mL respectively. Data are presented in heat-map format with blue representing no binding and yellow color representing the highest OD. Raw data were presented in bar-chart format below the corresponding heat-map. Results represent an average of two independent experiments. The regions of protein found to be important for antibody recognition are indicated in red above the heat map. (**C**) Sequences of the DENV E and ZIKV E peptides most strongly recognized by 1B-H1L1.

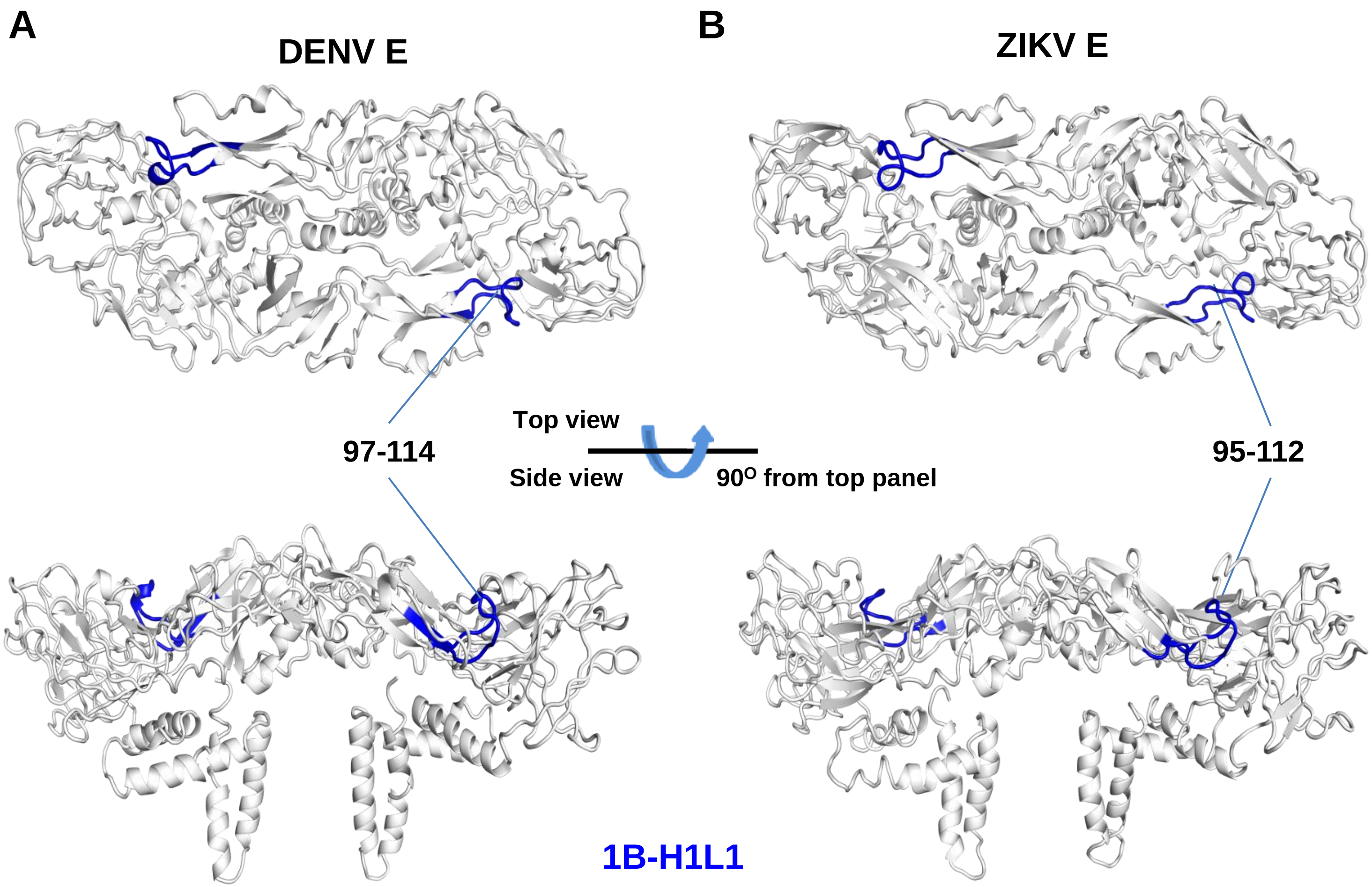

Supplemental Figure 7

Mapping of 1B-H1L1 epitope onto DENV E and ZIKV E structures. (**A**) Schematic diagrams showing the epitope position determined by peptide-ELISA in the DENV E glycoproteins based on the structural data retrieved from PDB records: 3J6U. (**B**) Schematic diagrams showing the epitope position determined by peptide-ELISA in the ZIKV E glycoproteins based on structural data retrieved from PDB records: 5IZ7. Peptide regions recognized by 1B-H1L1 are colored in blue.

Supplemental Figure 7 Kam *et al*., 2017

Supplemental Table 1
Characterization of human DENV mAbs against ZIKV.

Antibody	ZIKV binding capacity[a]	ZIKV neutralizing capacity (IC50)[b]
1D-H4L1	Medium	> 150 µg/mL
2C-H3L2	Medium	Non-neutralizing
2F-H1L1	Strong	> 150 µg/mL
5A-H6L1	Strong	Non-neutralizing
5B-H1L1	Medium	Non-neutralizing
5D-H1L2	Medium	Non-neutralizing
6C-H8L1	Medium	Non-neutralizing
8F-H1L1	Strong	Non-neutralizing
**1B-H1L1**	**Strong**	**19.25 µg/mL**
1D-H8L1	Strong	Non-neutralizing
**2F-H1L3**	**Strong**	**> 30 µg/mL**
**SIgN-3C**	**Medium**	**0.93 µg/mL**
3H-H1L1	Strong	> 150 µg/mL
4B-H2L1	Strong	> 150 µg/mL
5A-H1L1	None	Non-neutralizing
5D-H6L2	Strong	Non-neutralizing
6E-H1L1	Strong	> 150 µg/mL
7A-H1L1	Medium	Non-neutralizing
7E-H1L1	Medium	Non-neutralizing
7H-H1L1	None	Non-neutralizing
9B-H1L1	Medium	Non-neutralizing
9E-H2L2	Strong	Non-neutralizing
11E-H1L1	Strong	> 150 µg/mL

[a]Human DENV mAbs were grouped into strong binders, medium binders and non-binders according to the ZIKV virion-based ELISA results.

[b]Human DENV mAbs that did not show any *in vitro* ZIKV neutralizing activity at 30 µg/mL testing concentration were classified as “Non-neutralizing”. For mAbs showing neutralizing activity within the testing ranges (0.029 µg/mL – 30 µg/mL), nonlinear regression fitting was used to determine the IC50 values.
